# An integrative approach to phylogeography: investigating the effects of ancient seaways, climate, and historical geology on multi-locus phylogeographic boundaries of the Arboreal Salamander (*Aneides lugubris*)

**DOI:** 10.1186/s12862-015-0524-9

**Published:** 2015-11-04

**Authors:** Sean B. Reilly, Ammon Corl, David B. Wake

**Affiliations:** Museum of Vertebrate Zoology and Department of Integrative Biology, University of California, 3101 Valley Life Sciences Building, Berkeley, CA 94720-3160 USA

**Keywords:** Amphibians, Biogeography, California, Mitochondrial DNA, Nuclear loci

## Abstract

**Background:**

Phylogeography is an important tool that can be used to reveal cryptic biodiversity and to better understand the processes that promote lineage diversification. We studied the phylogeographic history of the Arboreal Salamander (*Aneides lugubris*), a wide-ranging species endemic to the California floristic province. We used multi-locus data to reconstruct the evolutionary history of *A. lugubris* and to discover the geographic location of major genetic breaks within the species. We also used species distribution modeling and comparative phylogeography to better understand the environmental factors that have shaped the genetic history of *A. lugubris.*

**Results:**

We found six major mitochondrial clades in *A. lugubris*. Nuclear loci supported the existence of at least three genetically distinct groups, corresponding to populations north of the San Francisco Bay and in the Sierra Nevada, in the Santa Cruz Mountains, and in the central coast and southern California. All of the genetic breaks in mitochondrial and nuclear loci corresponded to regions where historical barriers to dispersal have been observed in other species. Geologic or water barriers likely were the most important factors restricting gene flow among clades. Climatic unsuitability during glacial maximum may have contributed to the isolation of the mitochondrial clades in the central coast and southern California. A projection of our species distribution model to a future scenario with a moderate amount of climate change suggests that most of the range of *A. lugubris* will remain climatically suitable, but climatic conditions in the Sierra Nevada and low elevation areas in Southern California are likely to deteriorate.

**Conclusions:**

*Aneides lugubris* contains substantial cryptic genetic diversity as a result of historical isolation of populations. At least two (and perhaps three) evolutionarily significant units in *A. lugubris* merit protection; all six mitochondrial clades should be considered as management units within the species.

**Electronic supplementary material:**

The online version of this article (doi:10.1186/s12862-015-0524-9) contains supplementary material, which is available to authorized users.

## Background

Phylogeography has been widely used to study processes of diversification in the context of the geological and climatic history of a geographic region [1]. California has been the focus of a large number of phylogeographic studies, largely because of the great diversity of species inhabiting the state and the complex geological processes active in the region [2–4]. The California floristic province has been designated as one of the world’s top 25 hotspots of biodiversity and the state of California encompasses 70 % of this province [4, 5]. Endemism in the California floristic province is high, with 71 of the 584 (12 %) vertebrates in the region being endemics [5]. Phylogeographic studies of individual species in this region have often revealed high levels of phylogeographic structure [6–9] and the presence of cryptic species [10–13], which suggests that there are even greater levels of biodiversity in the region than previously recognized.

The California Floristic Province is home to 44 species of salamander, of which 33 are endemic to the province, according to the taxonomy in AmphibiaWeb (2015). The salamanders in this region have been the focus of many phylogeographic studies. Some studies have discovered additional cryptic species that have added to the endemism of the region [13–15]. Other studies have found high levels of divergence among geographically structured clades [6, 16, 17], many of which deserve protection as evolutionarily significant units [18]. The high levels of phylogeographic structure observed in these studies can be explained by the fact that salamanders in general have very low levels of dispersal. Salamanders may thus be particularly sensitive indicators of climatic and geologic factors that can lead to vicariance. Therefore, phylogeographic studies of salamanders are likely to be particularly informative about historical barriers to gene flow that have contributed to diversification in California.

Phylogeographic studies are especially effective when multiple forms of evidence are used to infer the history of a species. Mitochondrial DNA is widely used for phylogeography because it is particularly good at detecting historical relationships among populations, due to its low effective population size, high mutation rate, and lack of recombination [19, 20]. However, a gene tree based upon any single genetic region may not reflect the true history of a species because of incomplete lineage sorting or introgression [21]. One solution to this problem is to collect data for multiple loci, which allows researchers to utilize species tree methodologies for phylogenetics and can help control for any factors that only affect individual loci when inferring phylogeographic structure [22–24]. A second approach for interpreting phylogeographic patterns is to use species distribution models to generate hypotheses about where genetic breaks could have occurred due to historical range fragmentation in past climatic conditions [25, 26]. A third approach is to observe whether genetic breaks within a particular species are also found in other species, which would suggest that shared environmental barriers to gene flow have led to vicariance in a community of species [2, 27]. Our goal is to use a combination of multi-locus data, species distribution modeling, and comparative phylogeography to better understand the evolutionary history of a species of salamander endemic to the California floristic province, the Arboreal Salamander (*Aneides lugubris*).

**Fig. 1:**
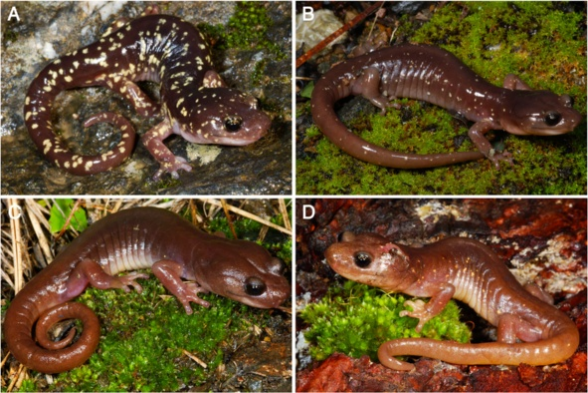
*Aneides lugubris* from (**a**) Lake Co., (**b**) Solano Co., (**c**) the Sierra Nevada Mtns., and (**d**) Santa Cruz Co. (Photos: Mitchell Mulks). Note the pronounced geographic variation in spotting among populations. Note also that the salamander in panel D has a head wound that likely resulted from agonistic interaction with another *A. lugubris*

**Fig. 2:**
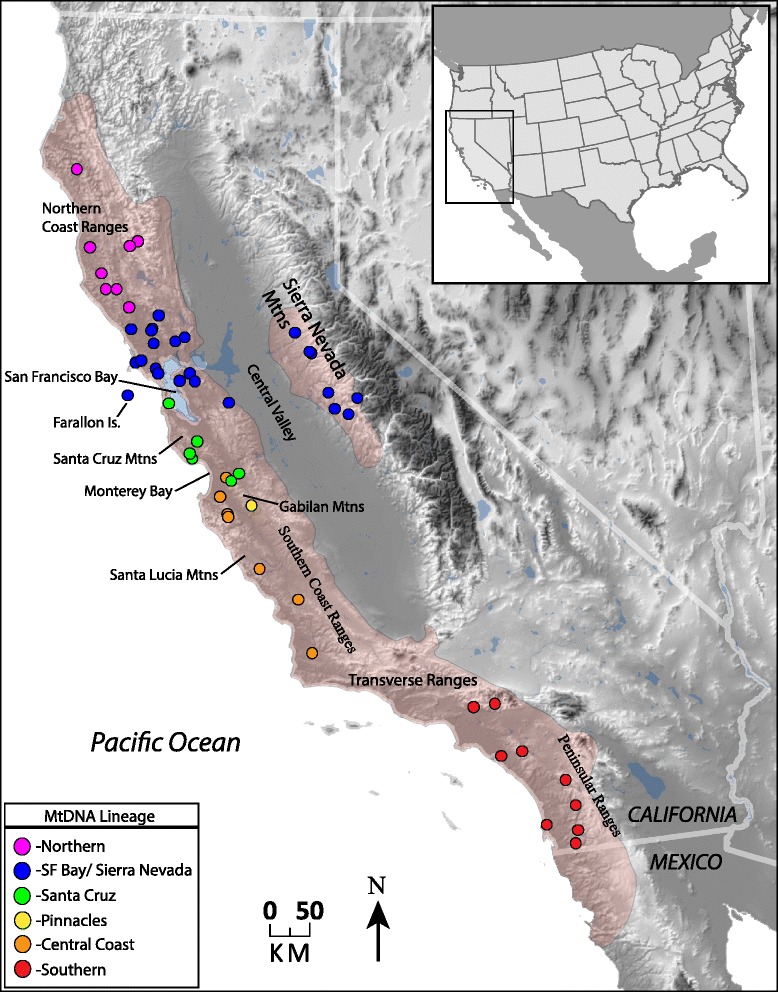
Relief map of California showing the estimated range of *Aneides lugubris* in beige, and the localities of genetic samples as circles. The colors of each sample locality correspond to the clade colors in Fig. 3

The Arboreal Salamander is a fully terrestrial, nocturnal, direct developing, lungless salamander in the family Plethodontidae [28]. The most common coloration is a brown or tan back with yellow spots, although the number and size of the spots vary between populations (Fig. 1). Arboreal Salamanders on South Farallon Island were previously recognized as a distinct subspecies *A. l. farallonensis* (Farallon Salamander; Van Denburgh, 1905 [29]), primarily on the basis of the presence of larger yellow spots than most mainland populations [30]. Arboreal salamanders are associated with oak woodland habitats, but they are also found in ecotonal regions and even treeless areas. They are well-adapted for climbing with well-developed limbs, a prehensile tail, and long toes. Arboreal Salamanders often deposit their eggs in holes of live oak trees and eggs have been found as high as 9 meters, and the species has been found as high as 18 meters in the canopy [31]. *Aneides lugubris* is unique among *Aneides*, and in fact among all plethodontid salamanders, in being hyperossified with many unique osteological features, and in being heavily muscularized [32]. The species occurs in the Coast Ranges from Humboldt County in northern California to a southern limit in the vicinity of Ensenada in Baja California Norte, Mexico, with disjunct populations in the western-central Sierra Nevada Mountains, on South Farallon Island, Año Nuevo Island, Catalina Island, and Los Coronados Islands off of northwestern Baja California (Fig. 2) [33, 34]. Despite its extensive range, *A. lugubris* has not yet been the focus of a comprehensive phylogeographic study. Some phylogeographic data were collected for *A. lugubris* as part of a study of nine codistributed Californian species [35]. This previous study examined relationships between county level units of *A. lugubris* using an unrooted mtDNA tree, but the sampling was limited and it did not take into account the possibility of multiple genetic clades occupying the same county [35]. In addition, an unpublished allozyme study suggested that the Sierra Nevada population is genetically distinct and that the Farallon Island population is most closely related to populations in nearby Marin County [36].

Phylogeographic study of *A. lugubris* is particularly relevant because of its potential for tracking the history of an important ecological assemblage in California. The complex and highly diversified California flora is derived from Tertiary formations and three are generally recognized: 1) plant formations of northern origin, widely distributed in a circum-polar pattern, the Arcto-Tertiary Geoflora, 2) formations that originated to the south, associated with the long trend of aridification in Mexico and southwestern United States, the Madro-Tertiary Geoflora, and 3) formations derived from tropical regions well to the south, the Neotropical Geoflora [37, 38]. Lowe (1950) developed a biogeographic hypothesis for species of *Aneides* grounded in the long history of the Arcto-Tertiary Geoflora [39]. All species of *Aneides*, except *A. lugubris*, are associated with coniferous and broad-leafed deciduous forests of northern origin. For example, species of *Aneides* in northwestern California are associated with redwood and mixed conifer forests, as well as with some broad-leafed associates such as Madrone, Tanbark Oak, Big-leaf Maple and Black Oak. *Aneides lugubris* is broadly sympatric with *A. flavipunctatus* and *A. vagrans* in southern Humboldt and western Mendocino counties. However, *A. lugubris* differs from its congeners in that its geographic distribution extends far to the south, well into regions dominated by Madro-Tertiary geofloral derivatives, in particular live oaks and sycamores. It is the only west coast species of *Aneides* found south of Monterey Bay, and in southern parts of its range, and on islands, it can be found in very open, unforested habitats. Thus, phylogeographic study of *A. lugubris* can provide insight into the processes that have shaped diversification of salamanders in the southern regions of California.

In this study, we analyzed the phylogeographic structure and population genetics of the Arboreal Salamander using both mitochondrial and nuclear sequence data with three main goals. First, we determined the number of distinct genetic clades, their geographic distributions, and their phylogenetic relationships in order to identify the major genetic breaks within the species. Second, we identified the potential causes of these biogeographic patterns by using species distribution modeling, comparative phylogeography, and considering the geological history of the region. Third, we considered the conservation implications of our data in terms of demarcating evolutionarily significant units and management units within the species [18, 40].

## Results

### Data characteristics

We obtained mtDNA data for 78 individuals (Fig. 2; Additional file 1: Table S1) and our dataset consisted of 1452 base pairs of aligned sequence data, with 884 bp of the *ND4* gene and 568 bp of the *cytb* gene. We collected nuclear data for 18 *A. lugubris* and 3 outgroup taxa and obtained sequences for five genes from all individuals except for one *A. lugubris* (MVZ213101), where low quality DNA prevented the collection of data for three nuclear loci. Our nuclear dataset contained a total of 48 variable sites.

### Phylogenetic analyses

**Fig. 3:**
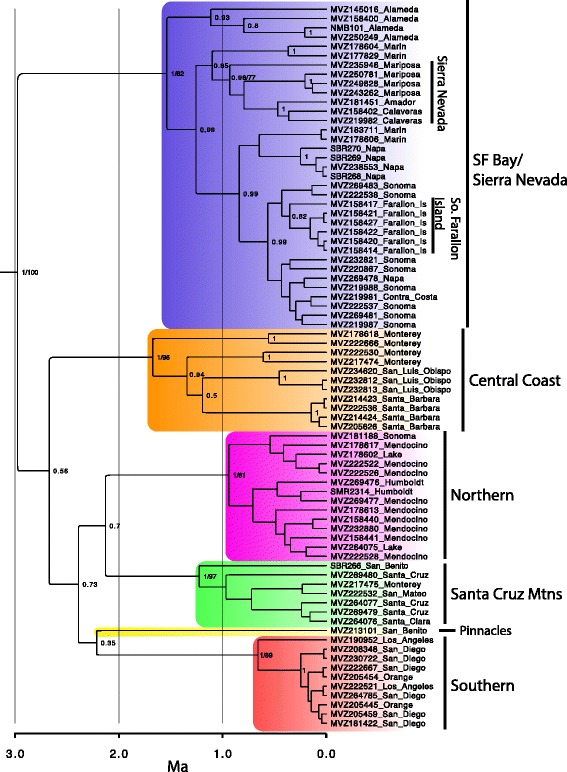
Time-calibrated Bayesian phylogeny of the *ND4* and *cytb* mitochondrial genes for *Aneides lugubris*. Numbers at nodes represent posterior probability support followed by bootstrap support after the backslash. The tree was pruned of the outgroup species to better visualize the relationships within *A. lugubris*

A time-calibrated Bayesian phylogenetic analysis conducted in BEAST [41] recovered six well-supported mtDNA clades (Fig. 3), which were also found in a similar analysis (Additional file 2: Figure S1) conducted with MrBayes [42, 43]. These include 1) a “Northern” clade, which occurs in northern Sonoma, Mendocino, Lake, and Humboldt counties, 2) a “SF Bay/Sierra Nevada” clade, which occurs in southern Sonoma, Marin, Napa, Contra Costa, and Alameda counties, as well as populations in the disjunct Sierra Nevada foothills and Farallon Islands, 3) a “Santa Cruz Mtns” clade, which occurs in San Mateo, Santa Cruz, Santa Clara, northeast Monterey, and northwest San Benito counties, 4) a single distinct “Pinnacles” sample from western central San Benito Co. in the vicinity of Pinnacles National Park, 5) a “Central Coast” clade, which inhabits the coast ranges of Monterey, San Luis Obispo, and Santa Barbara counties, and 6) a “Southern” clade from Los Angeles, Orange, and San Diego counties (Figs. 2 and 3). These clades are allopatrically distributed, except in the region where the Central Coast and Santa Cruz Mtns clades are parapatric, coming into close geographic contact and perhaps overlapping slightly. Furthermore, the Northern and SF Bay/Sierra Nevada clades come into relatively close geographic proximity in northern Sonoma Co., where further sampling may uncover a contact zone.

Our mtDNA phylogeny identified the probable source populations of the disjunct Sierra Nevada and Farallon Island populations. The Sierra Nevada samples form a monophyletic group that is sister to samples in Marin County. The Farallon Islands population forms a monophyletic group that is sister to samples from Central Sonoma County.

Both our Bayesian and maximum likelihood trees recovered the six clades with strong support. However, the relationships among the six clades were not well supported in the mtDNA trees. The six mitochondrial clades ranged from 2.1 % to 3.6 % sequence divergence from one another (Table 1). None of the clade-specific Tajima’s *D* statistics were significant, although all values were negative. We could not calculate Tajima’s *D* for the Pinnacles population because of inadequate sample size.

**Table 1:** Average uncorrected sequence divergence and Tajima’s *D* statistic between mitochondrial lineages based on the *ND4* gene

	Southern	Central Coast	Pinnacles	Santa Cruz	SF Bay/Sierra	Tajima's D	Sample Size
Southern	-	-	-	-	-	−1.73	10
Central Coast	0.036	-	-	-	-	−0.73	11
Pinnacles	0.030	0.027	-	-	-	N/A	1
Santa Cruz	0.031	0.034	0.024	-	-	−0.53	7
SF Bay/Sierra	0.021	0.025	0.023	0.028	-	−1.56	35
Northern	0.030	0.026	0.021	0.027	0.027	−1.35	14

### Species tree analyses

In order to generate a species tree, we had to assign our 18 individuals with nuclear sequence data to pre-specified groups before the analysis. In general, we did this by grouping samples that were in the same mtDNA clade. However, we deviated from this approach in two ways. First, we treated our Sierra Nevada sample as a distinct group, because we were interested in resolving the phylogenetic relationships of this disjunct population using data from both mtDNA and nuclear genes. Second, we treated the sample from northwest San Benito Co. (SBR266 from the Santa Cruz clade) as a distinct group because of its location at a contact zone.

**Fig. 4:**
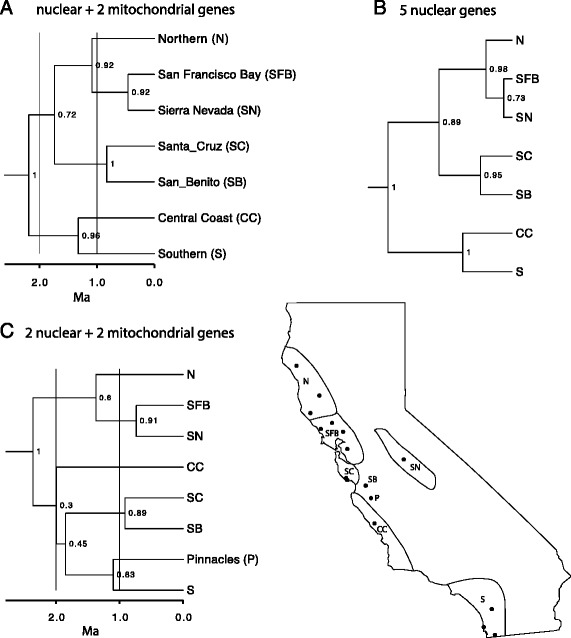
Species trees for *Aneides lugubris* using (**a**) two mtDNA + five nDNA loci, (**b**) five nDNA loci, and (**c**) two mtDNA + two nDNA loci. Sample localities of each clade can be seen on the map in the lower right corner. The trees were pruned of the outgroup species to better visualize the relationships within *A. lugubris*

Our species tree analyses, using all of our genetic data (5 nuclear and 2 mtDNA genes), and analyses that utilized only our nuclear loci, converged on the same tree topology (Fig. 4a, b). These multi-locus species tree analyses resolved the relationships among clades more fully than the mtDNA phylogeny. The basal split in both species trees occurred approximately 2.2 Ma, between a southern/central coast group and everything else (PP = 1, Fig. 4a, b). The Southern and Central Coast clades split ~1.3 Ma and are sister to each other with high support (PP > 0.95). The Northern clade split ~1.1 Ma from the clade containing the Sierra Nevada and SF Bay clades (mtDNA + nDNA PP = 0.92; nDNA PP = 0.98), which split from each other ~0.5 Ma. Both trees utilizing all nuclear genes recover a sister relationship between the Santa Cruz and San Benito groups (PP ≥ 0.95), which diverged ~0.8 Ma. Confidence intervals for major node ages from our tree utilizing all seven loci are found in Additional file 3: Table S2.

The genetically distinct Pinnacles sample could not be included in the above analyses because we were unable to obtain data for this population for three of the nuclear genes. Therefore, we analyzed a dataset that included two mtDNA plus two nuclear loci so that we could infer the relationship of our Pinnacles sample to the rest of the range. The resulting tree (Fig. 4c) finds the Pinnacles sample most closely related to the Southern clade, although support values were generally lower across the tree because of the reduced dataset.

### Genetic clustering analysis

**Fig. 5:**
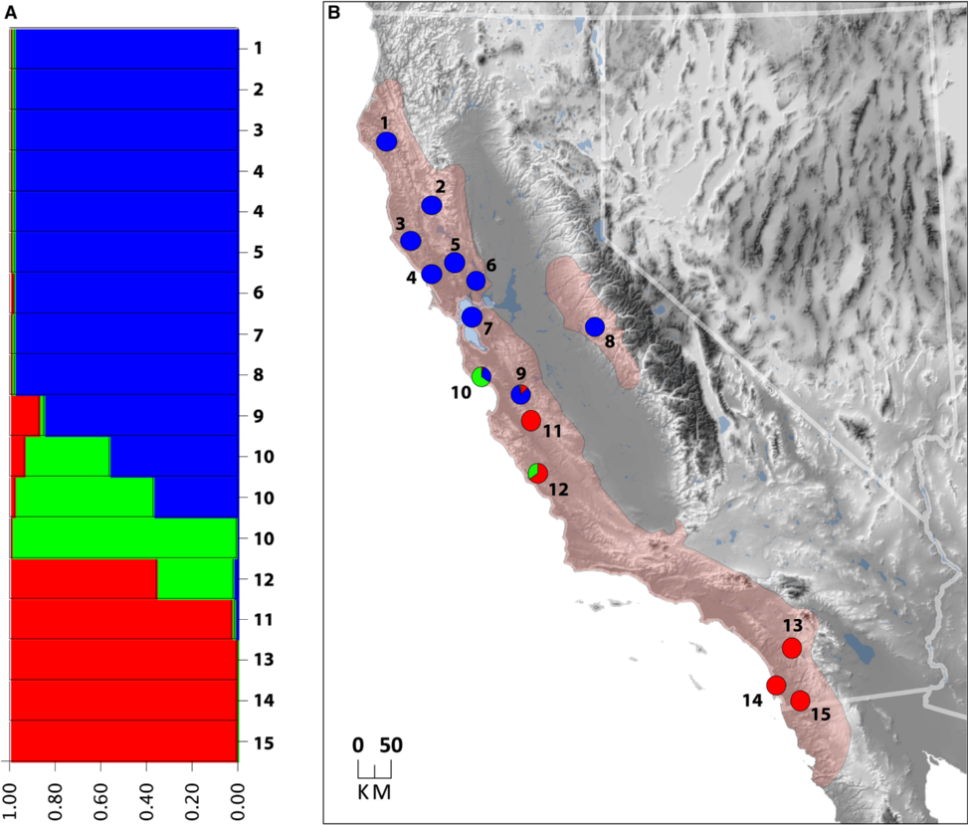
**a** Population structure of *Aneides lugubris* is depicted in the bar plot where each horizontal bar represents one individual, and the proportion of each color corresponds to the probabilities that the individual is assigned to one of three genetic clusters. **b** The spatial arrangement of these three gene pools where the number of each locality corresponds to the bar plot numbers, and the beige shaded area is the estimated range of *A. lugubris*

Our genetic clustering analysis of five nuclear genes supports the existence of three genetic clusters (Fig. 5). One includes all individuals assigned to the Northern and SF Bay/Sierra Nevada mtDNA clades (Fig. 5, blue points). A second is located in the Santa Cruz Mtns (green points), and a third is located in southern and central California (red points). Gene exchange appears to be in progress among all three genetic populations in the vicinity of the Monterey Bay when considering the mixed ancestry of some salamanders in the region.

### Species distribution modeling

**Fig. 6:**
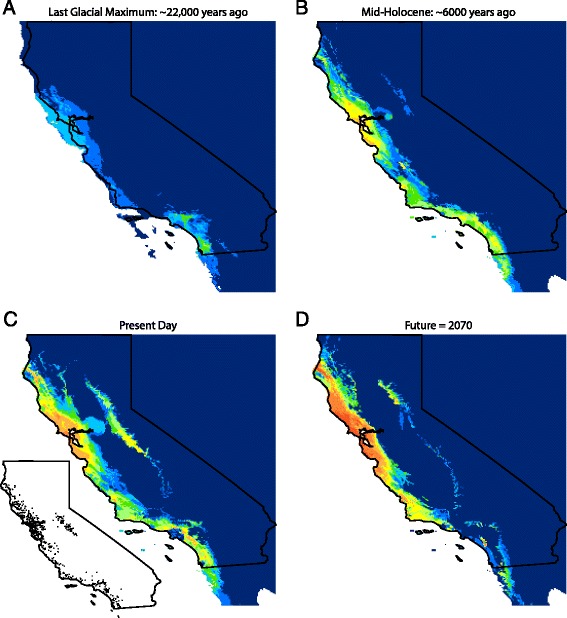
Species distribution models for *Aneides lugubris*. A species distribution model (panel **c**) was constructed using present climatic conditions and the locality points shown in the inset (both testing and training points shown). The distribution model was then projected to the climatic conditions of the last glacial maximum ~22,000 years ago (**a**) and the mid-Holocene ~6,000 years ago (**b**). The model was also projected to the inferred climatic conditions in the year 2070 under the Community Climate System Model 4 concentration pathway 4.5 global climate model (**d**). Colors to the red end of the spectrum indicate greater climatic suitability and colors to the blue end of the spectrum indicate lower climatic suitability

Our species distribution model (Fig. 6c) effectively captures the current occurrence of *A. lugubris,* as measured by the Area Under the Curve (AUC) values (Training data AUC = 0.950, test data AUC = 0.927). The six Bioclim variables with the highest permutation importance to the model were mean temperature of driest quarter, precipitation of coldest quarter, temperature annual range, mean temperature of the coldest quarter, minimum temperature of the coldest month, and annual mean temperature. In general, the distribution model closely matched the species occurrence data, but it also suggested two areas of potential occurrence where there are no species records. One area is in the Sierra Nevada, north of where the species is known to occur. The other area is in the Central Valley, between the San Francisco Bay and the Sierra Nevada.

A projection of the species distribution model to the climatic conditions of the last glacial maximum indicates a general reduction in favorable climatic conditions during this time (Fig. 6a). However, three potential refugia with relatively high climatic suitability are recognized, one in a broad area centered around the San Francisco Bay Area, another in southern California from Los Angeles to Baja California Norte, and the third in a region around San Luis Obispo. By the Mid-Holocene, the climate in the coastal areas where *A. lugubris* currently occurs was projected to be nearly as favorable as the present (Fig. 6b). However, conditions in the Sierra Nevada were still relatively poor.

A projection of the species distribution model to the climatic conditions in 2070 under a moderate climate change scenario suggests that favorable conditions will be maintained from the central coast to the northern part of the species distribution (Fig. 6d). However, climatic conditions are predicted to deteriorate in those portions of the Sierra Nevada where the species is known to occur. In addition, southern California is predicted to face poor climatic conditions for persistence, except in some of the mountainous areas.

## Discussion

Our phylogeographic analysis of *Aneides lugubris* defined six parapatrically distributed mtDNA clades that diverged from one another between ~1-2 million years ago (Figs. 3 and 4, Additional file 2: Figure S1). Mitochondrial DNA clades typically have abrupt geographic boundaries, but if species have limited dispersal abilities, such as *A. lugubris*, and especially if local populations are small, breaks are exaggerated [44] and may be less pronounced when using other markers. We tested our findings using nuclear loci, which supported the existence of at least three genetically distinct groups (Fig. 5); these generally correspond to (1) the SF Bay + Northern mtDNA clades, (2) the Santa Cruz Mountains clade, and (3) the Central Coast, Pinnacles, and Southern mtDNA clades (Fig. 2). The lack of fine-scale geographic structuring in the nuclear loci is likely due to their lower mutation rate and longer coalescence time than characteristic of mtDNA [19]. Analyses of nuclear loci may have also shown less genetic structure due to the influence of recent admixture among clades (Fig. 5). Although our analysis of the nuclear data was unable to corroborate the fine-scale genetic structure observed in mtDNA, nonetheless the data were very useful for reconstructing the phylogenetic relationships among the mtDNA clades (Fig. 4) and assessing gene flow among populations (Fig. 5). In addition, our species tree should give more accurate estimates of divergence times, because gene trees overestimate population divergence times and substantial heterogeneity can be found in the branch lengths of individual gene trees [22, 45]. Although extant genetic diversity within *A. lugubris* originated in the Pleistocene (Fig. 4), the lineage itself is much older, as evidenced by its split from the ancestor of *A. ferreus, A. vagrans,* and *A. flavipunctatus* ~20 million years ago [46]. We suspect that the origin of *A. lugubris* as a species is associated with ancestral dispersal into southern California, where the flora has been strongly influenced by Madro-Tertiary geofloral elements. If correct, the clades identified in our analysis reflect the origin of the species in the south and its subsequent dispersal into northern California. This hypothesis is supported by the fact that *A. lugubris* is the only west coast *Aneides* found south of Monterey Bay, the species is primarily associated with oak woodland habitat rather than coniferous forest, and the southern lineages are sister to all other lineages in our species tree.

Many phylogeographic studies of species in California have found high levels of cryptic genetic structure, similar to what we found in *A. lugubris* [2, 3, 9, 15]. Below we compare the geographic distribution of our mtDNA clades in relation to vicariance events, as well as to physical/climatic barriers to dispersal in other species. These comparisons serve as an additional way to verify that the major mtDNA clades were caused by vicariance and can contribute to a better understanding of the likely causes of genetic divergence. Overall, we find that the boundaries of the mtDNA clades correspond with historical barriers to dispersal and to the locations of vicariance in other species, which suggests that large-scale genetic structure in *A. lugubris* has likely been driven by historical isolation of populations.

### Comparative phylogeography

#### Monterey Bay

The deepest phylogeographic break for *A. lugubris* is located at the Monterey Bay area (Fig. 4). Many other members of the herpetofauna show a north–south break or range limits in this region including *Lampropeltis zonata* [47], *Emys marmorata* [48], *Ensatina* [6], *Batrachoseps* [12], *Aneides flavipunctatus* [34], *Ambystoma macrodactylum* [34], *Taricha rivularis* [49], *Taricha granulosa* [50], *Taricha* torosa [15], and *Dicamptodon ensatus* [34]. From the late Pliocene until the early Pleistocene (~2 Ma) this region was the site of a marine embayment extending into the Central Valley and dividing the Coast Ranges [51]. Uplift of the central Coast Ranges ~2 Ma closed off the embayment and created a large freshwater lake in the Central Valley, which was the source for a river that drained into the Monterey Bay until 600,000 years ago [52–55]. This river was likely a dispersal barrier for many taxa, including *A. lugubris*, and existed sufficiently long to facilitate genetic divergence in allopatry. Around 600,000 years ago, when the Central Valley began to drain into the San Francisco Bay, the Monterey Bay region ceased being a major water barrier. Salamanders such as *Taricha torosa* and *Batrachoseps gavilanensis* were then able to move north [12, 15]. However, this area was very low and probably remained inhospitable to movements across the area that is now the Pajaro River (which is presently a small river). There is some evidence of very local movement across the Pajaro River in *Ensatina* [6] and *Batrachoseps gavilanensis* [12], while other salamanders, including *Ambystoma macrodactylum*, *Aneides flavipunctatus*, *Batrachoseps attenuatus*, *Dicamptodon ensatus*, *Taricha granulosa*, and *Taricha rivularis* either have not dispersed or have failed to persist south of the Pajaro River [34], suggesting that the region remains a barrier to dispersal. In *A. lugubris,* the Monterey Bay area appears to be an area of genetic exchange among clades (Fig. 5), which shows that at least some dispersal has occurred in this area.

The marine embayment and Central Valley drainage are likely to be primarily responsible for the genetic break at the Monterey Bay area. However, the San Andreas Fault may also be involved, because the Central Coast and Southern Clades occur on the Pacific Plate, the Santa Cruz Mountains occur on a recently uplifted block of the Pacific Plate, and the SF Bay/Sierra and Northern Clades occur on the North American Plate. The Gabilan Mountains have moved northward rapidly on the left side of the San Andreas Fault, closing the gap where the drainage from the Central Valley to Monterey Bay previously occurred, and the Gabilan Range now reaches to the Pajaro River. However, samples from the inland portion of the Southern Coast Ranges are on the right side of the fault and located south of the Monterey embayment zone. Future sampling of the interior Southern Coast Ranges should determine if they are more closely related to the northern clades (Northern and SF Bay/Sierra), which would support the hypothesis that the San Andreas fault has contributed to this genetic break.

#### Transverse ranges

A phylogeographic break between the Southern and Central Coast populations is coincident with the Transverse Ranges, a rare example of an east–west trending mountain range within California. The Transverse Ranges are coincident with a phylogeographic break or range boundary for at least 35 different terrestrial animal species or species complexes [56], including the salamanders *Ensatina* [6], *Batrachoseps* [34], *Taricha torosa* [15], and *Ambystoma californiense* [34]. These mountains are likely to be a physical barrier to dispersal to many taxa, including *A. lugubris.* However, *A. lugubris* is found in many mountainous regions, so additional factors may contribute to the apparent barrier to dispersal. Our species distribution models indicate that the Transverse Ranges region had low climatic suitability to *A. lugubris* during the last glacial maximum (Fig. 6a). Glacial/interglacial cycles have been occurring for ~2.4 million years, since the beginning of the Quaternary Period [57], so the climatic conditions of glacial maxima likely affected the persistence of *A. lugubris* in this region at many time periods in the past. Fossils of *A. lugubris* are reported from multiple late Pleistocene deposits in southern California [58–60], suggesting a relatively stable distribution south of the Transverse Ranges.

#### Santa Cruz Mountains

One of our mtDNA clades is located in the Santa Cruz Mountains (Figs. 2 and 3) and populations in this geographic region also are differentiated in nuclear DNA (Fig. 5). The Santa Cruz Mountains are almost entirely west of the San Andreas Fault, on the Pacific Plate, and are geologically distinct from the surrounding Coast Ranges [61]. Populations in this area are bounded by the San Francisco Bay to the north, the Santa Clara Valley to the east, and the Monterey Bay to the south. Thus, both geologic and water barriers may have contributed to the isolation of the Santa Cruz populations. Many other species of salamander contain genetically distinct populations that are restricted to the Santa Cruz Mountains, including *Aneides flavipunctatus* [13], *Batrachoseps attenuatus* [7], and *Ambystoma macrodactylum* [34].

#### Pinnacles population

A single sample from the Pinnacles region of the Gabilan Mountains in central California is highly divergent in mtDNA, sufficient to warrant status as one of the major mtDNA clades (Fig. 3). This population has long been thought to be distinct because of its divergent coloration, consisting of very large yellow spots [30]. It was suggested that similarities in color pattern in a high frequency of individuals in populations of *A. lugubris* in the Pinnacles region and on South Farallon Island might have been due to shared ancestry [30]. Our genetic results do not support this hypothesis, so convergent evolution is likely responsible for large-spotted phenotypes shared by these two populations. The Pinnacles population is most closely related to salamanders from Southern California (Fig. 3, Fig. 4c, Fig. 5, population 11). A similar pattern has been found among species of slender salamander where *Batrachoseps gavilanensis*, found in the Gabilan Mountains, is more closely related to *Batrachoseps major major* in southern California than to *Batrachoseps nigriventis* in the central coast of California [62].

#### Sonoma/Mendocino break

The break between the SF Bay/Sierra Nevada and Northern mtDNA clades occurs near the border with Sonoma and Mendocino Counties (Fig. 2). This area contains a major phylogeographic break within *Aneides flavipunctatus* [13], hybrid zones between *Rana aurora* and *R. draytonii* [63], and *Dicamptodon ensatus* and *D. tenebrosus* [64], and the southern range limits of *Aneides vagrans*, *Rhyacotriton variegatus*, *Ambystoma gracile*, and *Ascaphus truei* [34].

In addition to differences in mtDNA, chromosomal differentiation is found in *A. lugubris* near the Sonoma/Mendocino break. Salamanders in Mendocino and Humboldt counties have a single pair of telocentric chromosomes (chromosome 13), while all other populations contain an additional pair of telocentric chromosomes at chromosome 12 [65]. These two populations apparently hybridize in southern and eastern Mendocino County, where salamanders are heteromorphic for chromosome 12 [65]. Although the chromosomal break appears to be in a similar position to the break between our mitochondrial “Northern” and “SF Bay” clades, finer scale sampling is needed to determine the relative positions of the two breaks. However, the observation that there is hybridization between these two chromosomal populations is in agreement with our population structure results (Fig. 5).

### Genetic divergence within major clades

#### Sierra Nevada

*Aneides lugubris* from the Sierra Nevada form a monophyletic clade most closely related to populations north of the San Francisco Bay in Marin County (Figs. 2 and 3). Our species distribution model predicts the presence of a corridor suitable for movement of salamanders between these two regions (Fig. 6c), although the climatic suitability is currently rather low. However, the populations in the Sierra Nevada diverged from the SF Bay clade approximately 0.4-0.5 Ma (Fig. 4a) and climatic conditions connecting the two regions may have been more suitable at that time. The pattern of a trans-valley leak from the Coast Ranges to the central Sierra Nevada across the Central Valley has been observed in many other species including *Batrachoseps attenuatus* [7], *Ensatina eschscholtzii xanthoptica* [6], *Contia tenuis* [10], *Lampropeltis zonata* [47], and California Turret Spiders [66].

A fossil *A. lugubris* reported from a late Miocene formation in the Sierra Nevada foothills in Stanislaus County dates to approximately 5 Ma [67]. Our results suggest that the Sierra Nevada population has only existed since the late Pleistocene, an estimate that was first proposed by Rosenthal [68], and subsequently supported by allozyme data [36]. It is likely that the late Miocene Sierra Nevada population went extinct and the current population is a result of Pleistocene dispersal across the Central Valley.

#### South Farallon Island

*Aneides lugubris* found on the South Farallon Island are most closely related to nearby populations in Sonoma County (Figs. 2 and 3). This result is similar to a previous allozyme study, which showed that the South Farallon Island population is closely related to salamanders in Marin County, just south of Sonoma [36]. The population of *A. lugubris* on the South Farallon Island likely dispersed over a historical land-bridge that formed between it and Sonoma/Marin Counties when sea level was lower during the last glacial maximum, as recently as ~20,000 years ago [69].

### Conservation implications

Phylogeographic information can be critical for decision-making relevant to conservation, because it can identify distinctive genetic units that may merit protection [10–13, 70]. While intraspecific genetic variation is important for the long-term viability of a species, distinct lineages may not meet the criteria of species status [18, 40, 71]. This has led to the concept of evolutionarily significant units (ESU), which have been defined as populations that are reciprocally monophyletic in mtDNA and which also exhibit substantial differences in allele frequencies at nuclear loci [18, 40]. In addition, there can be management units (MUs) within ESUs, which are populations that show significantly divergent mtDNA or nuclear DNA [18, 40]. The major genetic divide between northern and southern populations of *A. lugubris* (Figs. 4 and 5) shows that there are at least two ESUs within the species that warrant protection. The population in the Santa Cruz Mountains may be a third ESU (Figs. 3 and 5), but limited availability of fresh tissues from localities in this area prevented us from accurately determining the degree of distinctiveness and level of genetic isolation of the population. All six mtDNA clades warrant recognition as management units given their high levels of sequence divergence. While the Pinnacles population is divergent in mtDNA and has a distinctive color pattern compared to neighboring populations, the two nuclear loci sequenced for it were closely related to the Southern population. More sampling of localities in the Gabilan Mountains and interior Southern Coast Ranges along with data from multiple nuclear loci will be needed to determine the geographic range, genetic divergence, and conservation status of the Pinnacles population. Until future studies suggest otherwise, the South Farallon Island and Sierra Nevada populations should be conserved as management units due to their isolated nature and their monophyly in the mtDNA tree.

The analyses of population structure using nuclear genes (Fig. 5) suggest some gene flow when the northern and southern ESUs come into contact with each other and the Santa Cruz population. Given that the northern and southern ESUs do not show evidence of reproductive isolation, we recommend recognition of *A. lugubris* as a single taxon. Further studies focused on the Santa Cruz Mountains and Monterey Bay contact zones that include more localities and large-scale genomic datasets or microsatellites would be useful for determining the amount of gene flow in this area and assessing the species-level status of the three major gene pools detected in *A. lugubris.* While our mtDNA phylogeny shows that the South Farallon Island population (*A. lugubris farallonensis*) is monophyletic, this lineage is young compared to other lineages of *A. lugubris*. We suggest that the subspecies *A. l. farallonensis* be recognized only if other data (from morphological, ecological, or multi-locus genetic studies) should prove to be concordant with the mtDNA results.

The effects of global climate change are a major conservation concern for the persistence of species [72, 73]. Our species distribution model indicates that the majority of coastal regions inhabited by *A. lugubris* will remain climatically suitable (Fig. 6d), with the caveat that this projection was only for a moderate scenario of climate change. However, even with only moderate levels of climate change, *A. lugubris* could face inhospitable conditions in the southern and central Sierra Nevada and in low elevation areas in southern California. From a conservation standpoint, this finding is important for the management of the Southern Californian population, which is genetically distinct and has already been highly impacted by the loss of habitat due to anthropogenic changes. We recommend that efforts be made to monitor *A. lugubris* populations in San Diego, Orange, and Los Angeles Counties and that protection of montane habitats in this area may be crucial for the long-term persistence of populations in southern regions.

## Conclusions

The combination of multi-locus data, species distribution modeling, and comparative phylogeography offer a powerful approach for better understanding the evolutionary history of *A. lugubris*. The predominant causes of genetic breaks in *A. lugubris* likely were geologic or water barriers that prevented dispersal among populations, which led to allopatric divergence. Climatic unsuitability during glacial maxima likely contributed to the isolation between the Central Coast and Southern Californian mtDNA clades. We recommend that *A. lugubris* be maintained as a single species, because of evidence presented of nuclear gene flow in regions where mtDNA clades come into contact. At least two ESUs within *A. lugubris* merit protection; further genetic study is needed to assess whether populations in the Santa Cruz Mountains constitute a third ESU.

## Methods

### Genetic sampling

We sequenced the mitochondrial genes *ND4* and *cytochrome b* from 35 salamanders representing 26 localities. We also included *ND4* and *cytochrome b* sequences from GenBank for 43 salamanders from 27 additional localities [35]. Our total sampling strategy included 78 salamanders, representing 53 localities from 22 counties in California. Sample locality information can be found in Additional file 1: Table S1. Our sampling included *Aneides hardii*, *A. flavipunctatus*, *A. vagrans*, and *A. ferreus* as outgroups. GenBank numbers for all sequences used in genetic analyses can be found in Additional file 4: Table S3.

We sequenced a portion of the *ND4* mitochondrial gene and adjacent tRNAs (~880 bp) using the primers ND4 and LEU [74]. We also sequenced a portion of *cytb* (~385 bp) using the primers Cytb2 [75] and MVZ15 [76]. All reactions contained 18.3 μL water, 2.5 μL of 10X buffer, 1.5 μL magnesium chloride, 1.5 μL dNTPs (2 μM), 0.6 μL of each primer, 0.2 μL Taq polymerase, and 1 μL genomic DNA at concentration of 20–40 ng/μL. PCR products were cleaned by using ExoSAP-IT (USB, Cleveland, OH) before being labeled with fluorescent-dye nucleotides through cycle sequencing reactions for both forward and reverse primers. Ethanol precipitation was used to clean cycle sequencing products, which were then sequenced on an ABI 3730 sequencer (Applied Biosystems, Foster City, CA, USA). Raw sequence reads were combined in Codoncode Aligner 3.5.2 (CodonCode Corporation, Dedham, MA, USA) and aligned with MUSCLE [77]. The mitochondrial loci were concatenated and treated as a single locus for phylogenetic analyses.

We sequenced five nuclear loci for a subset of our samples that contained high quality DNA, which included 18 *Aneides lugubris* (representing 15 localities) and three outgroups (*Aneides flavipunctatus*-MVZ19969, *A. vagrans*-MVZ269484, and *A.ferreus*-MVZ269393). The nuclear loci that we sequenced were *CXCR4* (639 bp), *KIA* (534 bp), *RAG2* (904 bp), *SVEP1* (855 bp), and *ZEB* (821 bp), which gave a total of 3753 bp of nuclear sequence data. We used the primers for these loci designed by Shen et al. (2013) [78] and used their nested PCR approach (i.e. an initial PCR for the locus that is followed by a second PCR nested within the region amplified by PCR1). Our PCR reactions were 10 μl in total volume and contained 1X of PCR buffer, 25 mM MgCl_2_, 2 mM of each dNTPs, 5 μM of each primer, and 0.5 U Taq polymerase (Invitrogen), and 20–70 ng of genomic DNA. We followed a touchdown protocol to increase specificity during the PCR. Thermocycling began with an initial denature at 94 °C for 5 min, which was followed by 20 cycles of denature for 30 s at 94 °C, anneal for 30 s at a temperature that began at 60 °C and then decreased by 0.5 °C per cycle, and finally elongate for 1 min at 72 °C. The annealing temperature was then kept constant at 50 °C during 20 additional cycles. The PCR ended with a final hold at 72 °C for 5 min. To increase specificity, we diluted the PCR product from the first PCR reaction 1:10 before using it for the second PCR reaction. Sequencing and aligning of each nuclear gene followed the same protocol as listed above for mitochondrial loci. Allelic sequences were obtained from aligned nuclear genes using the program PHASE v2.1 [79, 80].

### Mitochondrial gene tree analyses

We utilized the software program BEAST v1.8 [41] to simultaneously estimate a Bayesian phylogeny and gene divergence times. We implemented the GTR + I + G model of sequence evolution as determined by jModelTest [81] for the concatenated mtDNA sequences and applied a lognormal relaxed clock model using a mutation rate of 0.8 % per million years as described for cytochrome b in the salamander *Taricha torosa* [82]. This mutation rate is comparable to the rate of *cytochrome b* as estimated across all salamanders (0.62 % + 0.16 %) [83]. We note that there are a number of sources of error that can affect the accuracy of a molecular clock [84] and that using an alternative rate would alter our time estimates, but we use this mutation rate to give a rough idea of the divergence times in *A. lugubris.* Two separate runs of 20 million generations were conducted, sampling every 1000 generations for a total of 20,000 saved trees per run. After checking for convergence by confirming that all effective sample size values were greater than 200 in TRACER v1.5 [85], we removed the first 2000 trees from each run and combined the remaining 36,000 trees in LOGCOMBINER [41, 86] to create a maximum clade credibility tree. We also ran MrBayes v3.2.2 [42, 43] under the GTR + I + G model with 4 heated chains for 20 million generations sampling every 2000 generations. After a burnin of 2 million generations the remaining 9000 trees were used to create a 50 % majority rule consensus tree (Additional file 2: Figure S1). The software program GARLI was used to create a maximum likelihood phylogeny using the default parameters [87]. Node support was assessed with 500 bootstrap replicates. Both Bayesian and maximum likelihood trees were rooted using the outgroups *Aneides hardii*, *A. flavipunctatus*, *A. vagrans*, and *A. ferreus*.

#### Mitochondrial data characteristics

We used the software program DNAsp v5 [88] to calculate the average uncorrected sequence divergence between each pair of our mitochondrial lineages. We used Tajima's *D* statistics to test for significant deviation from the expectations under a neutral model (no natural selection) with constant population size.

#### Species tree analysis

We used the program *BEAST [89] to estimate the relationships of the major clades recovered from our mtDNA phylogeny. We ran this program with three separate datasets: 1) five nuclear loci + two mitochondrial loci, 2) five nuclear loci, and 3) two nuclear loci + two mitochondrial loci. The first dataset is our preferred approach for resolving the relationships among clades, because it applies all of our data towards resolving the species tree. A combined analysis of mitochondrial and nuclear DNA is particularly powerful for finding the true species tree [24]. The second dataset of only nuclear loci was used to determine the extent to which the species tree was influenced by mtDNA, which could have a particularly strong influence given its relatively short coalescence time and rapid mutation rate. The third dataset was analyzed to include a geographically critical sample collected near Pinnacles in San Benito County (MVZ 213101), for which only 2 nuclear genes could be sequenced. Although not shown in Fig. 4, all trees were rooted using the outgroups *Aneides flavipunctatus*, *A. ferreus*, and A*. vagrans*, which are the three closest relatives to *A. lugubris* [46].

We used jModelTest [81] to determine the best fit model of sequence evolution for each gene, which specified the HKY model for *RAG2* and *ZEB*, the HKY + I model for *CXCR4, KIA,* and *SVEP1*, and the GTR + I + G model for the mtDNA. The general parameters that we used in *BEAST were a randomly generated starting tree, a Yule process for the species tree prior, and a piecewise linear and constant root population size model. For the two datasets that included mtDNA genes, we used a strict molecular clock setting with a rate of 0.8 % sequence divergence per million years for the mitochondrial DNA. Two runs of 100 million generations were completed for each of the three datasets and in each run one tree was sampled every 10,000 generations. We used Tracer [85] to verify that the two runs had converged, to choose the number of burnin generations so that we only used trees after stationarity had been achieved, and to ensure that the effective sample sizes for parameters were adequate. We removed the first 1000 saved trees of each run for the burnin and combined the saved trees from the two runs for a total of 18,000 saved trees per dataset. These two runs were combined to produce a maximum clade credibility tree for each of the three datasets.

#### Population structure

We ran our 5-gene nuclear dataset in the program STRUCTURE v2.3 [90] to estimate the number of distinct genetic populations and the amount of admixture between them. We ran the program using the linked model [91] where all variable sites within a locus are linked with the distance between them (in bp) specified. Ten runs for each population scheme were carried out from K = 1 to K = 10 with a burn-in of 50,000 steps followed by a 100,000 step run. The results were imported into the program STRUCTURE HARVESTER [92] and the most likely number of populations was determined by visualizing the DeltaK plot. The delta K plot and Ln P(D) plot can be found in the supplementary materials (Additional file 5: Figure S2). After determining the most probable population scheme, a final STRUCTURE run was carried out with the number of populations fixed and a burn-in of 500,000 steps followed by a run of 1,000,000 steps.

#### Species distribution modeling

We downloaded 4591 georeferenced locality data points for *A. lugubris* from the VertNet data portal (http://portal.vertnet.org/). We vetted the museum data by excluding points that were far outside the generally accepted range of the species (see [34]). Many data points were identical to other records and only a single representative locality was kept for the distribution model. In addition, some localities lacked climate data (such as small insular populations) and these were also excluded. We also used R [93] to generate a grid with cells 0.1 degrees on each side and randomly chose a single occurrence point per cell to avoid over fitting the model to areas with highly clustered occurrence points due to biased geographic sampling [94]. In the end, we had 400 localities that were used for our species distribution model.

Climate data were downloaded from Worldclim version 1.4 [95]. We downloaded 19 Bioclim variables at a resolution of 2.5 arc-minutes for past, present, and future climatic conditions. Data for past and future climatic conditions were generated from the Community Climate System Model 4 (CCSM4) global climate model. We downloaded data for the mid-Holocene (~6000 years ago) and the last glacial maximum (~22,000 years ago). Future climate projections were available for four different emissions scenarios at two different time points, but a full exploration of all these climate models was beyond the scope of this paper, which was focused on phylogeography. Therefore, we used just one of the future projections to provide a rough guide to locations that could be especially impacted by climate change. We used a climate projection for 2070, which is the average of climate projections for 2061–2080, and data for representative concentration pathway 4.5, which was one of the projections with a moderate amount of increased concentration of greenhouse gases.

We used the R packages ‘maptools’ and ‘rgdal’ when visualizing and manipulating the climate data [93, 96, 97]. We cropped all the climate data to a geographic span from the western and eastern borders of California (longitude −124.482 to −114.1312 degrees west) and from the northern border of California to just below the southern range limit of *A. lugubris* in Baja California Norte, Mexico (latitude 42.00952 to 31 degrees north). We then used the program Maxent version 3.3.3 k [98] to construct a species distribution model and to project it into past and future climatic conditions. Our Maxent runs used auto features, a logistic output, and randomly set aside 25 % of the data points for testing the model. We visualized the resulting output in ArcGIS 10.2.2.

## Availability of supporting data

DNA sequences have been deposited in GenBank and accession numbers can be found in Additional file 4: Table S3. DNA sequence alignments and phylogenetic trees from this study are available in the Dryad repository (doi:10.5061/dryad.kg4mq).

## Additional files

Additional file 1: Table S1. Museum voucher numbers, mtDNA clade designation, and locality information. (DOC 254 kb) Additional file 2: Figure S1. MrBayes phylogeny of the concatenated *ND4* and *cytb* mitochondrial genes. Numbers at nodes represent posterior probabilities. (DOC 638 kb) Additional file 3: Table S2. Divergence time estimates in millions of years (rounded to nearest 0.1 Ma) as estimated by *BEAST for our dataset containing 2 mtDNA and 5 nDNA genes. Clade abreviations: S = Southern, CC = Central Coast, SB = San Benito, SC = Santa Cruz, SFB = San Francisco Bay, SN = Sierra Nevada, N = Northern. (DOC 80 kb) Additional file 4: Table S3. GenBank numbers for sequences used in genetic analyses. *-sequence of less than 200 bp in length and of insufficient length to submit to GenBank. (DOC 212 kb) Additional file 5: Figure S2. STRUCTURE Harvester results from a comparison of K = 1 to K = 10 with ten replicates per population scheme. **a**) The Delta K value is based on the rate of change in the log probability of the data between successive values of the number of set populations (K) from 1–10. The largest Delta K value represents the most likely number of populations. **b**) The mean estimate of Ln P(D). **c**) The absolute value of the 2^nd^ order rate of change of the likelihood distribution (mean). **d**) The rate of change of the likelihood distribution (mean). (DOC 157 kb)
