# Supplementary material for: An integrative approach to phylogeography: investigating the effects of ancient seaways, climate, and historical geology on multi-locus phylogeographic boundaries of the Arboreal Salamander (Aneides lugubris)
Source: BMC Evol Biol. 2015 Nov 4;15:241. doi: 10.1186/s12862-015-0524-9 (PMC4632495; doi:10.1186/s12862-015-0524-9)
Supplement: Additional file 1: Table S1. — Museum voucher numbers, mtDNA clade designation, and locality information. (DOC 254 kb) [file 12862_2015_524_MOESM1_ESM.doc]

Supplementary Table 1. Museum voucher numbers, mtDNA clade designation, and locality information.

Supplementary Table 1 (cont.)
