# Supplementary material for: An integrative approach to phylogeography: investigating the effects of ancient seaways, climate, and historical geology on multi-locus phylogeographic boundaries of the Arboreal Salamander (Aneides lugubris)
Source: BMC Evol Biol. 2015 Nov 4;15:241. doi: 10.1186/s12862-015-0524-9 (PMC4632495; doi:10.1186/s12862-015-0524-9)
Supplement: Additional file 2: Figure S1. — MrBayes phylogeny of the concatenated ND4 and cytb mitochondrial genes. Numbers at nodes represent posterior probabilities. (DOC 638 kb) [file 12862_2015_524_MOESM2_ESM.doc]

Supplementary Figure 1. MrBayes phylogeny of the concatenated *ND4* and *cytb* mitochondrial genes. Numbers at nodes represent posterior probabilities.
