# Supplementary material for: An integrative approach to phylogeography: investigating the effects of ancient seaways, climate, and historical geology on multi-locus phylogeographic boundaries of the Arboreal Salamander (Aneides lugubris)
Source: BMC Evol Biol. 2015 Nov 4;15:241. doi: 10.1186/s12862-015-0524-9 (PMC4632495; doi:10.1186/s12862-015-0524-9)
Supplement: Additional file 3: Table S2. — Divergence time estimates in millions of years (rounded to nearest 0.1 Ma) as estimated by *BEAST for our dataset containing 2 mtDNA and 5 nDNA genes. Clade abreviations: S = Southern, CC = Central Coast, SB = San Benito, SC = Santa Cruz, SFB = San Francisco Bay, SN = Sierra Nevada, N = Northern. (DOC 80 kb) [file 12862_2015_524_MOESM3_ESM.doc]

Supplementary Table 2. Divergence time estimates in millions of years (rounded to nearest 0.1 Ma) as estimated by *BEAST for our dataset containing 2 mtDNA and 5 nDNA genes. Clade abreviations: S=Southern, CC=Central Coast, SB=San Benito, SC=Santa Cruz, SFB=San Francisco Bay, SN=Sierra Nevada, N=Northern.
