# Supplementary material for: An integrative approach to phylogeography: investigating the effects of ancient seaways, climate, and historical geology on multi-locus phylogeographic boundaries of the Arboreal Salamander (Aneides lugubris)
Source: BMC Evol Biol. 2015 Nov 4;15:241. doi: 10.1186/s12862-015-0524-9 (PMC4632495; doi:10.1186/s12862-015-0524-9)
Supplement: Additional file 4: Table S3. — GenBank numbers for sequences used in genetic analyses. *-sequence of less than 200 bp in length and of insufficient length to submit to GenBank. (DOC 212 kb) [file 12862_2015_524_MOESM4_ESM.doc]

Supplementary Table 3. GenBank numbers for sequences used in genetic analyses. *-sequence of less than 200 bp in length and of insufficient length to submit to GenBank.

Supplementary Table 3 (cont.)
