# Supplementary material for: An integrative approach to phylogeography: investigating the effects of ancient seaways, climate, and historical geology on multi-locus phylogeographic boundaries of the Arboreal Salamander (Aneides lugubris)
Source: BMC Evol Biol. 2015 Nov 4;15:241. doi: 10.1186/s12862-015-0524-9 (PMC4632495; doi:10.1186/s12862-015-0524-9)
Supplement: Additional file 5: Figure S2. — STRUCTURE Harvester results from a comparison of K = 1 to K = 10 with ten replicates per population scheme. a) The Delta K value is based on the rate of change in the log probability of the data between successive values of the number of set populations (K) from 1–10. The largest Delta K value represents the most likely number of populations. b) The mean estimate of Ln P(D). c) The absolute value of the 2nd order rate of change of the likelihood distribution (mean). d) The rate of change of the likelihood distribution (mean). (DOC 157 kb) [file 12862_2015_524_MOESM5_ESM.doc]

Supplementary Figure 2. STRUCTURE Harvester results from a comparison of K=1 to K=10 with ten replicates per population scheme. a) The Delta K value is based on the rate of change in the log probability of the data between successive values of the number of set populations (K) from 1-10. The largest Delta K value represents the most likely number of populations. b) The mean estimate of Ln P(D). c) The absolute value of the 2^nd^ order rate of change of the likelihood distribution (mean). d) The rate of change of the likelihood distribution (mean).
